# Supplementary material for: “I’m Hooked on e-cycling, I Can Finally Be Active Again”: Perceptions of e-cycling as a Physical Activity Intervention during Breast Cancer Treatment
Source: Int J Environ Res Public Health. 2023 Mar 15;20(6):5197. doi: 10.3390/ijerph20065197 (PMC10049330; doi:10.3390/ijerph20065197)
Supplement: Supplementary file 1 [file ijerph-20-05197-s001.zip › Supplementary Material S1.pdf]

Supplementary Materials

S1. <u>One-to-One Interview Guide</u>

<u>TOPIC GUIDE ONE</u>

<table>
<tr><td>Welcome; introduction; consent</td><td>- Thank you for taking part
- Remind participant that interview is recorded; start recording.
- Explain purpose of interview: to explore opinions relating to physical activity during cancer treatment, in particular an e-bike intervention
- Withdrawal without penalty (except 21 days after final interview)

HOW DID YOU HEAR ABOUT THIS STUDY?</td></tr>
<tr><td>Warm up</td><td>- Ask about day / plans for the week</td></tr>
<tr><td>Cancer Journey</td><td>If they are comfortable, discuss their cancer journey
o Just to check, would you be comfortable speaking to me about your cancer journey in slightly more detail than the initial survey?

- Tell me about your cancer journey
o How far along [first diagnosis, relapse etc.]?
o Undergoing treatment [how long for / left to go]?
o If treatment ended, how long ago?

- Explain to me your current physical health
o Daily energy levels
o Cancer ‘commitments’</td></tr>
<tr><td>Physical Activity</td><td>What does your current physical activity engagement look like?

o Do you participate in regular physical activity?
o How often? Is it consistent each week?
o Preferred physical activity modes?

- Describe what your physical activity engagement looked like across your lifespan [briefly]?

- How has your physical activity engagement changed throughout your cancer journey?</td></tr>
</table>

	- Explain to me what motivates you to engage in physical activity?<br>o Skip if no physical activity completed<br><br>- Do you think engaging in PA during cancer treatment is important?<br>o Do you think it impacts your recovery?<br><br>- Explain, if any, your perceived **barriers** to physical activity?<br>o **Cancer related**<br>o Non-cancer related (if deemed important / relevant)<br><br>- Explain, if applicable, how your cancer has interfered with your ability to do physical activity?<br>o **Energy levels**<br>o Specific cancer / treatment side effects
Bike / E-bike usage	Describe to me your current understanding of an e-bike<br>o What is it?<br>o What they're used for?<br>o Benefits / limitations compared to normal cycling?<br>o Do you know anyone with an e-bike? If so, what is their perception?<br><br>**Explain what an e-bike is**<br>- An e-bike is an electronic bike<br>o bicycles with a battery-powered "assist" that comes via pedalling<br>o Can help when cycling up hills<br>o Make pedalling easier<br>- Some come with a motor by the press of a button<br>- Research has found people cycle further and more regularly (Castro et al., 2019)<br><br>Describe your current cycling engagement<br>- Do you cycle? If so, how often?<br>- **Have you previously used an e-bike or regular bicycle?**<br>o Do you own one or loaned one?<br><br>Why did you agree to take part in this research?<br><br>Initial thoughts / opinions of e-cycling<br>- Does it interest you?<br>- Perceived ease of use during cancer treatment<br>- E-cycling vs conventional cycling?

	Ask for any **generic comments** about e-cycling
E-cycling taster session	Discuss the process of the 1-hour e-cycling taster session<br><br>- Arrange a date / time if not already done so<br>- Explain that more information will be provided nearer the time
Final comments and thank you	- Please specify any other comments you wish to add?<br>- Thank you for your time<br>- I hope you enjoy your e-cycling taster session!

<u>TOPIC GUIDE TWO</u>

Welcome; introduction; consent	- Thank you for taking part<br>- Remind participant that interview is recorded; **start recording.**<br>- Explain purpose of interview: to explore opinions relating to physical activity during cancer treatment, in particular an e-bike intervention<br>- Withdrawal without penalty (except 21 days after final interview)
Warm up	- Ask about day / plans for the week
E-cycling taster session	Question the participant about their 1-hour taster session<br><br>- Tell me about the session<br>- **Particular likes / dislikes?**<br>- How did you find riding the e-bikes?<br>- E-bike compared to conventional bike?<br><br>**Did you have to plan the session around any treatments?**
E-bike Intervention	From your experiences using an e-bike:<br><br>- Can you identify any potential benefits of using an e-bike during your cancer journey?<br><br>- **Describe whether you perceive e-cycling to be feasible during your cancer journey?**

	- Would you have a preference for an e-bike rather than a regular bicycle?<br>- If yes/no, why?<br>- Would an e-bike be easy enough to use during:<br>- times of high stress<br>- lacking energy<br>- other barriers to cycling<br>- **Potential reasons for not engaging?**<br><br>Cancer journey<br>- Is / was there a time in your cancer journey that you would perceive most difficult to engage in a physical activity intervention?<br>- When?<br>- Why?<br>- More specifically, an e-bike intervention?<br>- **In these 'difficult' times, what is the biggest barrier to exercise?**<br>- **How does e-cycling compare to other modes of physical activity you have done previously?**<br>- Describe a time during your cancer journey that would be feasible to engage in an e-bike intervention?<br>- Pre-treatment? During treatment? Post-treatment? At what point post-treatment?<br>- If you had access to an e-bike would you consider using it instead of a car or public transport.<br>- Under what circumstance?<br>- Would you use it for commuting for chores/shopping?<br>- Would you use it for leisure PA?<br>- Would you use it for anything else?<br>- Do you think you would like to monitor your e-cycling activity?<br>- If so, would a paper diary or activity tracking watch be better?<br>- Interviewer to give a brief overview of what an e-bike intervention might involve. Explain whether an e-bike intervention sounds like something you would be interested in trying.<br>- If yes/no, why?<br>- What would you want from the study?<br>- What would motivate you to take part?<br><br>Speak freely about the intervention in general

Final comments and thank you	- Please specify any other comments you wish to add?<br>- Thank you for taking part
